# Early loss to follow-up of recently diagnosed HIV-infected adults from routine pre-ART care in a rural district hospital in Kenya: a cohort study

**DOI:** 10.1111/j.1365-3156.2011.02889.x

**Published:** 2011-09-30

**Authors:** Amin S Hassan, Katherine L Fielding, Nahashon M Thuo, Helen M Nabwera, Eduard J Sanders, James A Berkley

**Affiliations:** 1 KEMRI/Wellcome Trust Centre for Geographic Medicine Research (Coast)Kilifi, Kenya; 2 London School of Hygiene and Tropical MedicineLondon, UK; 3 Centre for Clinical Vaccinology & Tropical Medicine, University of OxfordOxford, UK

**Keywords:** HIV, lost to follow-up, retention, pre-antiretroviral therapy

## Abstract

**Objective:**

To determine the rate and predictors of early loss to follow-up (LTFU) for recently diagnosed HIV-infected, antiretroviral therapy (ART)-ineligible adults in rural Kenya.

**Methods:**

Prospective cohort study. Clients registering for HIV care between July 2008 and August 2009 were followed up for 6 months. Baseline data were used to assess predictors of pre-ART LTFU (not returning for care within 2 months of a scheduled appointment), LTFU before the second visit and LTFU after the second visit. Logistic regression was used to determine factors associated with LTFU before the second visit, while Cox regression was used to assess predictors of time to LTFU and LTFU after the second visit.

**Results:**

Of 530 eligible clients, 178 (33.6%) were LTFU from pre-ART care (11.1/100 person-months). Of these, 96 (53.9%) were LTFU before the second visit. Distance (>5 km *vs.* <1 km: adjusted hazard ratio 2.6 [1.9–3.7], *P* < 0.01) and marital status (married *vs.* single: 0.5 [0.3–0.6], *P* < 0.01) independently predicted pre-ART LTFU. Distance and marital status were independently associated with LTFU before the second visit, while distance, education status and seasonality showed weak evidence of predicting LTFU after the second visit. HIV disease severity did not predict pre-ART LTFU.

**Conclusions:**

A third of recently diagnosed HIV-infected, ART-ineligible clients were LTFU within 6 months of registration. Predictors of LTFU among ART-ineligible clients are different from those among clients on ART. These findings warrant consideration of an enhanced pre-ART care package aimed at improving retention and timely ART initiation.

## Introduction

During the past decade, there has been a substantial roll out of HIV/AIDS services in sub-Saharan Africa (sSA), where an estimated 24 million people are infected (UNAIDS 2010). A critical barrier to effective scale up of these services is attrition of patients from care. The main component of attrition has been identified as loss to follow-up (LTFU) (Rosen *et al.* 2007).

Most studies assessing LTFU in HIV programmes have observed patients from initiation of antiretroviral therapy (ART) and report high rates of early attrition and mortality (Tassie *et al.* 2010; Brinkhof *et al.* 2008; Lawn *et al.* 2008; Rosen *et al.* 2007). A review of ART programmes in sSA found rates of LTFU ranging from 20% at 6 months to nearly 40% at 2 years after ART initiation (Rosen *et al.* 2007). The main risk factors for LTFU are lower baseline body mass index (BMI), lower CD4 count, lower haemoglobin, WHO stage III/IV, younger patients and being men (Amuron *et al.* 2009; Bassett *et al.* 2009; Brinkhof *et al.* 2008; Toure *et al.* 2008; Ochieng-Ooko *et al.* 2010). These data suggest that LTFU from ART programmes is mainly associated with advanced HIV disease. Moreover, most deaths among patients on ART occur in the early months after treatment initiation, which has been attributed to late access to ART and consequent severe immunosuppression (Lawn *et al.* 2008; Brinkhof *et al.* 2008; Bassett *et al.* 2010; Boulle *et al.* 2008; Fenner *et al.* 2010; Russell *et al.* 2010).

Strategies to improve follow-up generally focus on bringing lost patients back into the healthcare system through tracing. However, for example, in Zambia’s national treatment programme, more than two thirds of patients who had dropped out of care could not be contacted, even after several attempts (Krebs *et al.* 2008). A systematic review of outcomes of patients lost from HIV care and treatment programmes in resource-limited settings found that 20–60% of patients who could be traced had died (Brinkhof *et al.* 2009). As tracing patients is time-consuming, expensive and often unsuccessful, LTFU from HIV care remains a major challenge.

A better understanding of pre-ART LTFU is critical to designing interventions aimed at improving long-term care and timely initiation of ART. Few studies have exclusively assessed pre-ART LTFU in Africa (Larson *et al.* 2010; Bassett *et al.* 2010; Amuron *et al.* 2009; Losina *et al.* 2010; Lessells *et al.* 2011). Of these, only one study from South Africa has assessed factors associated with retention in patients who were not eligible for ART at enrolment into HIV care (Lessells *et al.* 2011). It is unclear whether their findings are generalizable to other regions in sSA with differing HIV prevalence, services and social context.

In this study, we aim to determine the rate of early LTFU among adults who were recently diagnosed with HIV but not yet eligible for ART and to identify baseline predictors associated with pre-ART LTFU in a rural district hospital in Kenya.

## Methods

### Study site

The study was conducted at the Comprehensive Care and Research Clinic (CCRC) at Kilifi District Hospital, a public healthcare institution located in the coastal province of Kenya. HIV care at the CCRC is provided according to the National AIDS and STI Control Program guidelines, which are largely adopted from the WHO guidelines.

Clients routinely undergo rapid voluntarily or provider-initiated HIV testing at clinical departments or other entry points within the hospital. HIV-infected clients are referred to the CCRC for registration into HIV care. CD4 cell count and haemoglobin investigations are routinely requested at registration and every 6 months thereafter or if otherwise clinically indicated. Newly registered clients are immediately prescribed daily cotrimoxazole prophylaxis, and a 2-week appointment is given to assess for side effects, have a CD4 count done if not done at registration and to discuss laboratory results. Clients meeting the WHO eligibility criteria (WHO 2006) are initiated on ART and followed up monthly thereafter. Those not immediately eligible for ART continue to receive cotrimoxazole prophylaxis and are subsequently monitored at 2-month intervals.

In 2008, two additional programmes were initiated in the clinic: a randomized controlled trial examining the potential benefits of routine empiric de-worming of HIV-1-infected clients who do not yet qualify for ART [‘Anti Helminthic Trial (AHT)’, clinicaltrials.gov NCT00507221] and a ‘Food By Prescription (FBP)’ programme, sponsored by the World Food Programme where clients from poor socio-economic backgrounds with susceptibility for malnutrition are enrolled and benefit from monthly rations of cereals, flour and cooking oil. Active tracing of defaulting clients was instituted for those enrolled in both programmes.

### Study design

A prospective, hospital cohort was designed to follow up pre-ART adults (≥15 years old), registering for HIV care from 1st July 2008 to 31st August 2009, and who were not yet eligible for ART. We define pre-ART as the period from registration to ART treatment initiation. Clients were followed up for 6 months after their registration at the CCRC. Exclusions included clients who were diagnosed with HIV infection more than 3 months before registration into care. Ineligibility for ART, an inclusion criterion for the study, was determined by (i) CD4 count ≥200 cells/μl, (ii) WHO stage 1 or 2 in the absence of a CD4 count or (iii) clients who did not yet have a CD4 count or WHO staging at baseline.

### Exposure variables

Upon registration at the CCRC, socio-demographic data were collected and entered into an electronic data system. At every follow-up visit, anthropometry and clinical data were captured. Immunological data were captured upon receipt of laboratory results.

**Figure 1:**
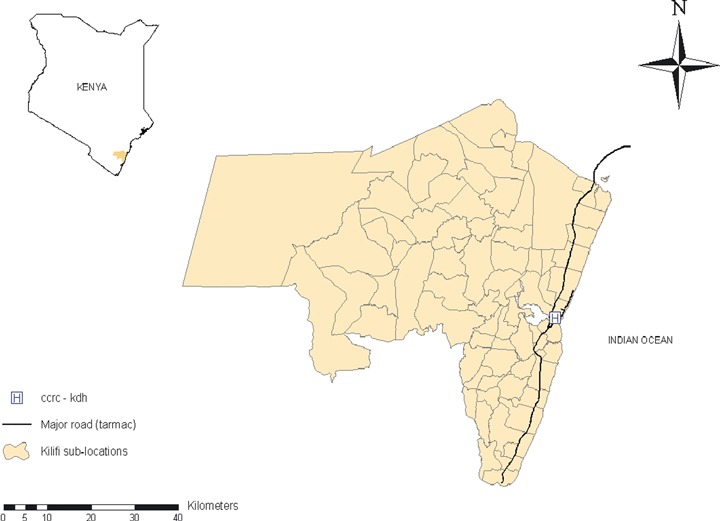
A map showing the location of the Comprehensive Care and Research Clinic (CCRC) within the Kilifi District Hospital (KDH), and the main road linking the hospital to other parts of the district.

Data on population density at the sub-location level were obtained from the Kenyan national population census. Sub-locations were categorized into densely populated and sparsely populated, using the median population density as the cut-off threshold. Distances between sub-locations where clients resided and the hospital, and the shortest distance from the sub-locations to the main road leading to the hospital were estimated using ArcInfo (ArcCatalog version 9.2, Esri, UK) (Figure 1).

Season at registration and at dropout was determined from the dates at registration and last clinic appointment, respectively. These were stratified to wet seasons (March–June, October–November) and dry seasons (December–February, July–September).

### Outcome variables

Prior studies have used various definitions of LTFU (Fox & Rosen 2010; Losina *et al.* 2010; Tassie *et al.* 2010; Yiannoutsos *et al.* 2008; Yu *et al.* 2007). Data on a multisite HIV treatment cohort in Lusaka, Zambia, were used to determine an empirical ‘days-late’ definition of LTFU among clients on ART. Their analyses suggest that 60 days since the last appointment was the best fitting definition of LTFU (Chi *et al.* 2010).

The primary outcome of this study was pre-ART LTFU over 6 months of follow-up after first registration into HIV care. We defined ‘LTFU’ as clients who were more than 60 days late for a scheduled appointment and did not return within the follow-up period. We further divided this into two end points of interest: (i) Clients who registered for HIV care but did not return over the given follow-up period (referred henceforth as ‘LTFU before the second visit’), and (ii) Clients who made at least one follow-up visit after registration but were subsequently LTFU within the follow-up period (referred henceforth as ‘LTFU after the second visit’).

To facilitate survival analysis, we assumed clients LTFU before the second visit contributed 1 day of follow-up each. All clients who were in follow-up 6 months after registration were censored at 6 months. Clients who were known to have died or transferred care to other health facilities or LTFU were censored at their last attended visit date.

Clients initiated on ART during the follow-up period were censored at the date they started ART. AHT and FBP clients were also censored at their dates of enrolment into either of the two programmes, whichever came first, as these programmes included active tracing of defaulters.

### Statistical methods

Means and standard deviations (SD) were used to present data following a normal distribution. Median and interquartile ranges were used to present data that were not normally distributed. Cross-tabulation of baseline categorical data with AHT/FBP was made to describe their distributions. Correlation between variables was assessed using the Pearson correlation coefficient.

Cox regression analysis was used to determine predictors of time to LTFU and time to LTFU after the second visit. Lexis expansion was adopted to assess the hazard of LTFU based on changing seasons by splitting the follow-up period into wet and dry seasons. The Kaplan–Meier method was used to estimate the survival probability of being LTFU from HIV care by 6 months of follow-up time. Logistic regression analysis was used to determine factors associated with LTFU before the second visit. A forward stepwise model building approach was used in all the analyses. Predictors with a likelihood ratio test *P*-value of <0.10 were considered and presented in the multivariable regression models. Analyses were carried out using Stata statistical software (Stata Intercooled version 11; StataCorp, College Station, TX, USA).

### Ethical considerations

Scientific and ethical approval was granted by the Kenya Medical Research Institute, Scientific Steering Committee, National Ethical Review Committee (No. 1341) and the Ethics Committee of the London School of Hygiene and Tropical Medicine, University of London. Participants provided written informed consent.

## Results

### Cohort characteristics

**Figure 2:**
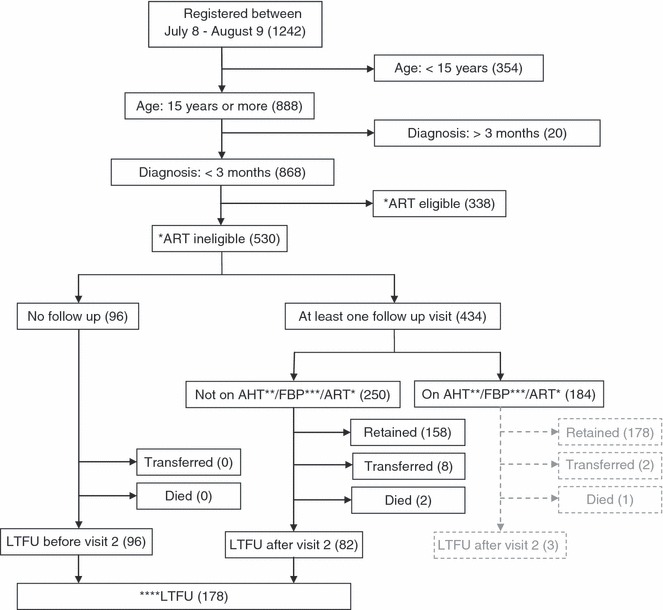
Diagram showing the flow of HIV infected adults registered and followed up for routine HIV care for 6 months in a district hospital in Kenya (*N* = 1242). *Antiretroviral Therapy (ART), **Anti Helminthic Trial (AHT), ***Food By prescription (FBP), ****Lost to follow up (LTFU).

Of the 1242 clients registering for care at the CCRC between July 2008 and August 2009, 868 were adults aged ≥15 years recently diagnosed with HIV. Of these, 530 (61.1%) were not eligible for ART at enrolment and were included in this analysis. During the 6-month follow-up period, 184 of these clients enrolled in the AHT [61 (11.5%)] and/or FBP [75 (14.2%)], while 89 (16.8%) started ART; 178 (33.6%) were LTFU, of whom 96 (53.9%) were LTFU before the second visit (Figure 2).

Of the 530 clients included in the analysis, 118 (22.3%) were men (Table 1). At registration, there were no substantial differences in the baseline characteristics between clients who were later enrolled in the AHT and/or FBP programmes compared to those who were not: age (mean 32.6 *vs.* 32.3 years, *P* = 0.834), BMI (mean 20.9 *vs.* 21.3 kg/m^2^, *P* = 0.254), haemoglobin levels (mean 10.2 *vs.* 10.2 g/dl, *P* = 0.949) and WHO staging (WHO stage II: 40.6%*vs.* 47.6%, *P* = 0.179). However, clients who were enrolled in the AHT/FBP programmes had higher baseline CD4 counts than those who were not (mean 486.8 *vs.* 431.4 cells/ul, *P* = 0.015).

**Table 1:** Distribution of baseline characteristics of newly diagnosed HIV-infected antiretroviral therapy ineligible adults registered for routine HIV care in a district hospital in Kenya [frequency (column %), *N* = 530]

Risk factor	Categories	On AHT/FBP		
		Yes (*n* = 128)	No (*n* = 402)	Total (*n* = 530)
Gender	Male	17 (13.3)	101 (25.1)	118 (22.3)
	Female	111 (86.7)	301 (74.9)	412 (77.7)
Age (years)*	Mean (SD)	32.6 (10.8)	32.3 (10.1)	32.4 (10.2)
	(Min–Max)	(16.8–78.2)	(15.1–75.0)	(15.1–78.2)
Age group (years)	15.0–25.0	31 (24.2)	98 (24.4)	129 (24.3)
	25.1–35.0	54 (42.2)	172 (42.8)	226 (42.6)
	>35.0	43 (33.6)	132 (32.8)	175 (33.0)
Marital status	Single	11 (8.6)	55 (13.7)	66 (12.5)
	Married (monogamous/polygamous)	81 (63.3)	263 (65.4)	344 (64.9)
	Separated/Divorced/Widowed	36 (28.1)	84 (20.9)	120 (22.6)
Entry point†	In-patient wards	13 (10.2)	72 (17.9)	85 (16.1)
	Out-patient/VCT centers	115 (89.8)	330 (82.1)	445 (83.9)
Religion	Christian	82 (64.1)	254 (63.2)	336 (63.4)
	Muslim	16 (12.5)	82 (20.4)	98 (18.5)
	Others	30 (23.4)	66 (16.4)	96 (18.1)
Education status	No schooling	45 (35.2)	113 (28.1)	158 (29.8)
	Primary schooling	69 (53.9)	207 (51.5)	276 (52.1)
	Secondary/Higher	14 (10.9)	82 (20.4)	96 (18.1)
Population density (sub-location level)	Sparsely populated (<25 people/km^2^)	62 (48.4)	169 (42.0)	231 (43.6)
	Densely populated (>25 people/km^2^)	62 (48.4)	167 (41.5)	229 (43.2)
	Missing	4 (3.1)	66 (16.4)	70 (13.2)
Distance from home to the hospital (km)*	Mean (SD)	10.2 (9.2)	11.8 (10.5)	11.4 (10.2)
	(Min–Max)	(0.8–37.6)	(0.8–44.5)	(0.8–44.5)
Group distance from home to the hospital (km)	<5.0	49 (38.3)	138 (34.3)	187 (35.3)
	5.0–20.0	50 (39.1)	136 (33.8)	186 (35.1)
	>20.0	25 (19.5)	111 (27.6)	136 (25.7)
	Missing	4 (3.1)	17 (4.2)	21 (4.0)
Distance from home to the main road (km)*	Mean (SD)	3.2 (5.1)	3.4 (5.5)	3.4 (5.4)
	(Min–Max)	(0.0–22.3)	(0.0–41.7)	(0.0–41.7)
Group distance from home to the road (km)	<1.0	66 (51.6)	194 (48.3)	260 (49.1)
	1.0–5.0	31 (24.2)	87 (21.6)	118 (22.3)
	>5.0	27 (21.1)	104 (25.9)	131 (24.7)
	Missing	4 (3.1)	17 (4.2)	21 (4.0)
Season at registration	Dry	77 (60.2)	227 (56.5)	304 (57.4)
	Wet	51 (39.8)	175 (43.5)	226 (42.6)
WHO staging	Stage I	76 (59.4)	182 (45.3)	258 (48.7)
	Stage II	52 (40.6)	165 (41.0)	217 (40.9)
	Missing	0 (0.0)	55 (13.7)	55 (10.4)
BMI (kg/m^2^)*	Mean (SD)	20.9 (3.8)	21.3 (3.7)	21.2 (3.7)
	(Min–Max)	(13.5–35.4)	(14.5–38.7)	(13.5–38.7)
BMI groups (kg/m^2^)	<18.5	37 (28.9)	62 (15.4)	99 (18.7)
	≥18.5	91 (71.1)	276 (68.7)	367 (69.3)
	Missing	0 (0.0)	64 (15.9)	64 (12.1)
CD4 count (cells/ul)*	Mean (SD)	486.8 (184.0)	431.4 (205.8)	450.3 (200.1)
	(Min–Max)	(206.0–1276.0)	(200.0–1100.0)	(200.0–1276.0)
CD4 groups (cells/ul)	200–350.0	25 (19.5)	105 (26.1)	130 (24.5)
	350.1–500.0	43 (33.6)	54 (13.4)	97 (18.3)
	>500.0	49 (38.3)	66 (16.4)	115 (1.7)
	Missing	11 (8.6)	177 (44.0)	188 (35.5)
Hemoglobin (g/dl)^*^	Mean (SD)	10.2 (2.0)	10.2 (2.3)	10.2 (2.2)
	(Min–Max)	(5.3–15.4)	(4.8–18.0)	(4.8–18.0)
Hemoglobin groups (g/dl)	<8.0	18 (14.1)	29 (7.2)	47 (8.9)
	8.0–10.0	25 (19.5)	48 (11.9)	73 (13.8)
	10.1–12.0	36 (28.1)	51 (12.7)	87 (16.4)
	>12.0	18 (14.1)	26 (6.5)	44 (8.3)
	Missing	31 (24.2)	248 (61.7)	279 (52.6)

AHT/FBP, Anti Helminthic Trial/Food By Prescription.

* Mean [standard deviation (SD)] and [Minimum/Maximum (Min–Max)] included for continuous variables.

† Site where clients have been referred from, Body Mass Index (BMI), Voluntary Counseling and Testing (VCT), World Health Organization (WHO).

The average distance from clients’ homes to the hospital was 11.4 (SD 10.2) km and from home to the main road, 3.4 (SD 5.4) km. Direct distance to the hospital was correlated with near distance to the main road (Pearson correlation coefficient, 0.75) and was excluded from further analysis based on the argument that patients living far from hospital but near the main road had better access to the hospital than those living far from the main road, other factors held constant. We were unable to estimate the distance from some clients as sub-location data were either missing 21 (4.0%) or the clients lived outside the district 49 (9.2%).

### Predictors of time to LTFU

**Figure 3:**
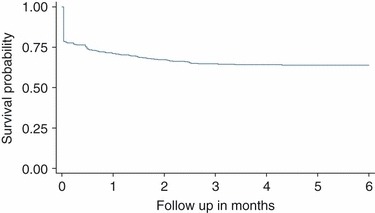
Kaplan–Meier curve showing loss to follow-up of recently diagnosed HIV infected adults from routine pre-antiretroviral therapy care, followed up for 6 months in a district hospital in Kenya (*N* = 530).

Overall, 530 newly diagnosed HIV-infected adults contributed 1606 person-months of observation (pmo). Of these, 178 (33.6% [95% CI: 29.6–37.6]) were LTFU, giving a rate of 11.1 (9.6–12.8)/100 pmo (Figure 3). In multivariable analyses, marital status and distance independently predicted pre-ART LTFU (Table 2).

**Table 2:** Cox univariable and multivariable analysis for predictors of pre-antiretroviral therapy ‘LTFU’ in newly diagnosed HIV infected adult clients registered for routine HIV care in a district hospital in Kenya (*N* = 530)

Risk factors	Categories	LTFU, *n*/100 pmo *n* = 178	KM survival probability†	Cox univariable analysis			Cox multivariable analysis (*n* = 509)		
				Crude HR	95% CI	*P*-value*	Adjusted HR‡	95% CI	*P*-value*
Gender	Male	43/3.7	0.62	1.0	–				
	Female	135/12.4	0.65	0.9	0.6–1.3	0.52	–	–	–
Age group (years)	15.0–25.0	52/3.6	0.59	1.0	–				
	25.1–35.0	73/6.9	0.66	0.8	0.5–1.1				
	>35.0	53/5.5	0.68	0.7	0.5–1.0	0.19	–	–	–
Marital status	Single	34/1.6	0.46	1.0	–		1.0	–	
	Married (monogamous/polygamous)	104/10.9	0.68	0.5	0.4–0.8		0.5	0.3–0.6	
	Separated/Divorced/Widowed	40/3.5	0.65	0.6	0.4–1.0	0.01	0.5	0.3–0.8	<0.01
Entry point	In-patient wards	38/2.2	0.54	1.0	–				
	Out-patient /VCT centers	140/13.8	0.67	0.7	0.5–0.9	0.03	–	–	–
Religion	Christian	112/10.0	0.65	1.0	–				
	Muslim	27/3.4	0.71	0.8	0.5–1.2				
	Others	39/2.6	0.57	1.3	0.9–1.8	0.18	–	–	–
Education status	No schooling	47/5.0	0.68	1.0	–				
	Primary schooling	92/8.4	0.66	1.1	0.8–1.6				
	Secondary/Higher	39/2.7	0.57	1.4	0.9–2.2	0.28	–	–	–
Population density (sub-location level)	Sparsely populated (<25 people/km^2^)	64/7.5	0.71	1.0	–				
	Densely populated (>25 people/km^2^)	63/7.5	0.71	1.0	0.7–1.4	0.93	–	–	–
Distance from home to the road (km)	<1.0	65/9.1	0.74	1.0	–		1.0	–	
	1.0–5.0	35/3.6	0.69	1.3	0.8–1.9		1.3	0.9–2.0	
	>5.0	71/2.7	0.43	2.6	1.8–3.6	<0.01	2.6	1.9–3.7	<0.01
Season at registration	Dry	108/9.3	0.63	1.0	–				
	Wet	70/6.8	0.68	0.9	0.6–1.2	0.39	–	–	–
WHO staging§	I	65/8.8	0.72	1.0	–				
	II	59/7.1	0.72	1.1	0.8–1.5	0.62	–	–	–
BMI groups (kg/m^2^)§	<18.5	26/3.0	0.70	1.0	–				
	≥18.5	92/12.7	0.73	0.9	0.6–1.4	0.77	–	–	–
CD4 groups (cells/ul)§	200–350.0	14/5.3	0.88	1.0	–				
	350.1–500.0	6/3.9	0.94	0.6	0.2–1.5				
	>500.0	17/4.2	0.82	1.5	0.7–2.9	0.12	–	–	–
Hemoglobin groups (g/dl)§	<8.0	6/1.8	0.86	1.0	–				
	8.0–10.0	6/2.8	0.90	0.7	0.2–2.1				
	10.1–12.0	12/3.4	0.85	1.1	04–2.9				
	>12.0	5/1.6	0.86	1.0	0.3–3.2	0.81	–	–	–
Time updated season¶	Dry	105/8.5	0.63	1.0	–				
	Wet	73/7.6	0.67	0.9	0.7–1.2	0.40	–	–	–

pmo, person months of observation; CI, confidence interval; KM, Kaplan–Meier.

* Likelihood Ratio Test *P*-value.

† Kaplan–Meier Survival probabilities at 6 months of follow up.

‡ Adjusted for other variables, Body Mass Index (BMI), Voluntary Counseling and Testing (VCT), World Health Organization (WHO).

§ Missing data: WHO staging [*n* = 55 (10.4%)], BMI [*n* = 64 (12.1%)], CD4 count [*n* = 188 (35.5%)], Hemoglobin [*n* = 279 (52.6%)].

¶ Time varying covariate expanded to assess for the hazard of LTFU over changing seasons.

Compared to single clients, clients who were married at registration into HIV care had half the rate of being LTFU (adjusted hazard ratio, aHR [95% CI], *P*-value; 0.5 [0.3–0.6], *P* < 0.01). Clients living more than 5 km from the main road were more likely to be LTFU than those living within 1 km of the road (2.6 [1.9–3.7], *P* < 0.01).

### Predictors of LTFU before the second visit

Of the 530 recently HIV-diagnosed adults, 96 (18.1% [95% CI: 14.8–21.4]) were LTFU before the second visit. In multivariable analysis, distance and marital status were independently associated with LTFU before the second visit (Table 3).

**Table 3:** Logistic univariable and multivariable analyses for predictors of ‘LTFU before the second visit’ in newly diagnosed HIV infected adult clients registered for routine HIV care in a district hospital in Kenya (*N* = 530)

Risk factors	Categories	LTFU (%) *n* = 96	\Logistic univariable analysis			Logistic multivariable analysis (*n* = 509)		
			Crude OR	95% CI	*P*-value*	Adjusted OR†	95% CI	*P*-value*
Gender	Male	25/118 (21.2)	1.0	–				
	Female	71/412 (17.2)	0.8	0.5–1.3	0.33	–	–	–
Age group (years)	15.0–25.0	32/129 (24.6)	1.0	–				
	25.1–35.0	33/226 (14.6)	0.5	0.3–0.9				
	>35.0	31/175 (17.8)	0.7	0.4–1.1	0.06	–	–	–
Marital status	Single	23/66 (34.9)	1.0	–		1.0	–	
	Married (monogamous/polygamous)	51/344 (14.8)	0.3	0.2–0.6		0.2	0.1–0.5	
	Separated/Divorced/Widowed	22/120 (18.3)	0.4	0.2–0.8	<0.01	0.3	0.1–0.6	<0.01
Entry point	In-patient wards	23/85 (26.7)	1.0	–				
	Out-patient/VCT centers	73/445 (16.4)	0.5	0.3–0.9	0.02	–	–	–
Religion	Christian	61/336 (18.2)	1.0	–				
	Muslim	14/98 (14.3)	0.8	0.4–1.4				
	Others	21/96 (21.9)	1.3	0.7–2.2	0.39	–	–	–
Education status	No schooling	24/158 (15.2)	1.0	–				
	Primary schooling	54/276 (19.5)	1.4	0.8–2.3				
	Secondary/Higher	18/96 (19.0)	1.3	0.7–2.5	0.51	–	–	–
Season at registration	Dry	53/304 (17.5)	1.0	–				
	Wet	43/226 (19.0)	1.1	0.7–1.7	0.64	–	–	–
Population density (sub-location level)	Sparsely populated (<25 people/km^2^)	30/229 (8.6)	1.0					
	Densely populated (>25 people/km^2^)	22/231 (20.0)	1.4	0.8–2.6	0.23	–	–	–
Distance from home to the road (km)	<1.0	23/260 (8.9)	1.0	–		1.0	–	
	1.0–5.0	21/118 (17.7)	2.2	1.2–4.2		2.8	1.4–5.4	
	>5.0	49/131 (37.4)	6.2	3.5–10.7	<0.01	7.0	3.9–12.6	<0.01
WHO staging‡	Stage I	20/258 (7.7)	1.0	–				
	Stage II	25/217 (11.5)	1.5	0.8–2.9	0.16	–	–	–
BMI groups (kg/m^2^)‡	<18.5	8/99 (8.1)	1.0	–				
	≥18.5	36/367 (9.8)	1.2	0.6–2.8	0.60	–	–	–

LTFU, loss to follow-up.

* Likelihood Ratio Test *P*-value.

† Adjusted for other variables, Body Mass Index (BMI), Voluntary Counseling and Testing (VCT), World Health Organization (WHO).

‡ Missing data: WHO staging [*n* = 55 (10.4%)], BMI [*n* = 64 (12.0%)].

Clients living more than 5 km from the main road had the greatest odds of being LTFU before the second visit compared to those living within a kilometre from the road (adjusted odds ratio, aOR [95% CI], *P*-value; 7.0 [3.9–12.6], *P* < 0.01). Being married at the time of registration into HIV care was associated with an 80% reduction in the odds of being LTFU before the second visit compared to being single (0.2 [0.1–0.5], *P* < 0.01). Immunological predictors were not assessed in this analysis as only two of the 96 clients LTFU before the second visit had a CD4 count performed at registration.

### Predictors of time to LTFU after the second visit

We restricted this analysis to those clients who made at least one follow-up visit after registration into HIV care (*n* = 434). Of these, 82 (18.9% [95% CI: 15.2–22.6]) were determined LTFU after the second visit, giving a rate of 5.1 (4.1–6.4)/100 pmo. Education status, distance from the main road and time updated season showed weak evidence of predicting LTFU after the second visit in multivariable analysis (Table 4).

**Table 4:** Cox univariable and multivariable analysis for predictors of Pre-antiretroviral therapy ‘LTFU after the second visit’ in newly diagnosed HIV infected adult clients registered for routine HIV care in a district hospital in Kenya (*N* = 434)

Risk factors	Categories	LTFU, *n*/100 pmo *n* = 82	Cox univariable analysis			Cox multivariable analysis (*n* = 416)		
			Crude HR	95% CI	*P*-value*	Adjusted HR†	95% CI	*P*-value*
Gender	Male	18/3.7	1.0	–				
	Female	64/12.4	1.0	0.6–1.7	0.98	–	–	–
Age group (years)	15.0–25.0	20/3.6	1.0	–				
	25.1–35.0	40/6.9	1.0	0.6–1.7				
	>35.0	22/5.5	0.7	0.4–1.3	0.37	–	–	–
Marital status	Single	11/1.6	1.0	–				
	Married (monogamous/polygamous)	53/10.9	0.8	0.4–1.4				
	Separated/Divorced/Widowed	18/3.5	0.8	0.4–1.6	0.70	–	–	–
Entry point	In-patient wards	15/2.2	1.0	–				
	Out-patient/VCT centers	67/13.8	0.7	0.4–1.3	0.29	–	–	–
Religion	Christian	51/10.0	1.0	–				
	Muslim	13/3.4	0.8	0.4–1.5				
	Others	18/2.6	1.3	0.8–2.3	0.37	–	–	–
Education status	No schooling	23/5.0	1.0	–		1.0	–	
	Primary schooling	38/8.3	1.0	0.6–1.7		1.0	0.6–1.7	
	Secondary/Higher	21/2.7	1.6	0.9–3.0	0.17	2.0	1.1–3.6	0.05
Population density (sub-location level)	Sparsely populated (<25 people/km^2^)	42/7.5	1.0	–				
	Densely populated (>25 people/km^2^)	33/7.5	0.8	0.5–1.2	0.30	–	–	–
Distance from home to the road (km)	<1.0	42/9.1	1.0	–		1.0	–	
	1.0–5.0	14/3.6	0.9	0.5–1.6		0.9	0.5–1.7	
	>5.0	22/2.7	1.6	0.9–2.7	0.08	1.7	1.0–3.0	0.08
Season at registration	Dry	55/9.3	1.0	–				
	Wet	27/6.8	0.7	0.4–1.1	0.08	–	–	–
WHO staging‡	I	45/8.8	1.0	–				
	II	34/7.1	0.9	0.6–1.4	0.72	–	–	–
BMI groups (kg/m^2^)‡	<18.5	18/3.0	1.0	–				
	≥18.5	56/12.7	0.8	0.5–1.4	0.46	–	–	–
CD4 groups (cells/ul)‡	200–350.0	13/5.3	1.0	–				
	350.1–500.0	6/3.9	0.6	0.2–1.6				
	>500.0	16/4.2	1.5	0.7–3.1	0.16	–	–	–
Hb groups (g/dl)‡	<8.0	6/1.8	1.0	–				
	8.0–10.0	5/2.8	0.6	0.2–1.8				
	10.1–12.0	11/3.4	1.0	0.4–2.7				
	>12.0	5/1.6	1.0	0.3–3.2	0.70	–	–	–
Time updated season§	Dry	52/8.5	1.0	–		1.0		
	Wet	30/7.6	0.7	0.4–1.1	0.09	0.7	0.4–1.0	0.09

pmo, person months of observation; CI, confidence interval.

* Likelihood Ratio Test *P*-value.

† Adjusted for other variables, Loss to follow-up (LTFU), Body Mass Index (BMI), Voluntary Counseling and Testing (VCT), World Health Organization (WHO).

‡ Missing data: WHO staging [*n* = 4 (0.9%)], BMI [*n* = 12 (2.8%)], CD4 count [*n* = 94 (21.7%)], Hemoglobin [*n* = 185 (42.6%)].

§ Time varying covariate expanded to assess for the hazard of drop out over changing season.

Clients with secondary/higher education at registration into HIV care had twice the rate of being LTFU after the second visit compared to those with no formal schooling (aHR [95% CI], *P*-value; 2.0 [1.1–3.6], *P* = 0.05). Similarly, clients living more than 5 km from the main road were more likely to be LTFU after the second visit compared to those living within a kilometre from the road (1.7 [1.0–3.0], *P* = 0.08). Clients also had a 30% lower rate of being LTFU after the second visit in the wet seasons compared to the dry seasons (0.7 [0.4–1.0], *P* = 0.09).

## Discussion

Our findings from a routine HIV care clinic in a rural district hospital in Kenya suggest that a third of recently diagnosed HIV-infected clients registered for pre-ART care were LTFU within 6 months of registration. More than half of those who were LTFU did not return for follow-up HIV care in the 6 months after registration. Distance and marital status at registration into HIV care independently predicted LTFU. Distance and marital status were also independently associated with LTFU before the second visit, while distance, level of education at registration into HIV care and seasonality independently predicted LTFU after the second visit.

Longer distances from health facilities reduce accessibility as clients have to spend more money on travel and take more time away from work. Although HIV services are mostly offered free of charge, indirect costs are a deterrent to retention of clients in care. Longer distance, long travel time and high costs of transport are major barriers to the access of HIV care (Amuron *et al.* 2009; Ochieng-Ooko *et al.* 2010; Maskew *et al.* 2007; Losina *et al.* 2010). It is possible that a small number of clients may also have opted for HIV care in more accessible peripheral clinics without notifying the CCRC of their transfer.

Single clients were more likely to be LTFU from HIV care immediately after registration. This may be because single clients do not have a support person, hence more likely to be negatively affected by HIV-related stigma. This has been shown to be an important barrier to adherence and retention in care (Merten *et al.* 2010; McGuire *et al.* 2010). Most single people are also conventionally young, and young age has been found to be a risk factor for LTFU, albeit in patients on ART (Karcher *et al.* 2007; Ochieng-Ooko *et al.* 2010). However, age was not found to be an independent predictor of pre-ART LTFU in our setting.

Interestingly, level of education at registration into HIV care had a weak association with LTFU after the second visit, suggesting that better-educated clients were likely to return after registration for follow-up visits but dropout thereafter. A plausible explanation is that educated clients have better-paying jobs, and may opt to acquire the main pre-ART intervention, the cheap and readily available cotrimoxazole, over the counter to avoid the HIV-related stigma of being seen in the clinic.

We also found weak evidence of an association between dry seasons and LTFU after the second visit. Given that the community is mainly agrarian, some clients may be forced to seek alternative socio-economic activities to sustain their livelihoods during the dry seasons. This may necessitate working long hours or out-migration to other districts in search of jobs.

Importantly, HIV disease severity as determined by lower CD4 count, lower haemoglobin levels, lower BMI and late clinical staging did not predict pre-ART LTFU in this setting. Most previous studies on loss to HIV care in clients on ART have identified these factors as independently associated with LTFU. Our findings, together with recent data from South Africa (Losina *et al.* 2010), suggest that the dynamics and risk factors for pre-ART retention differ considerably from those found among clients who have started ART.

In view of the fact that literature suggests high rates of early mortality after ART initiation in Africa (Lawn *et al.* 2008; Brinkhof *et al.* 2008; Bassett *et al.* 2010), it is plausible that recently diagnosed HIV-infected clients register for care and dropout while they are still healthy, only to present later with advanced HIV disease necessitating immediate ART initiation. If this is the case, then we argue that focusing and redirecting resources towards provision of an enhanced standard package of pre-ART care may improve timely initiation of ART and influence early adverse outcomes.

The pre-ART package of care may include a structured framework of counselling and support at both testing and registration into HIV care. This approach has been applied in ART programmes to enhance retention and ART adherence in different settings with relative success (Etienne *et al.* 2010). Evidently, the same approach is equally important in pre-ART clients registering for HIV care.

Other pre-ART care services may include provision of prophylactic anthelmintics, isoniazid preventive therapy (IPT), multivitamins and nutritional support in form of food programmes. These interventions may serve as an incentive for follow-up and counter the indirect costs incurred.

Studies on anthelminthic drugs and IPT have shown that these cheap and readily available interventions administered in pre-ART clients have the potential to slow HIV disease progression (Walson *et al.* 2008; Grant *et al.* 2005). Hence, an improved pre-ART package of care may serve not only to enhance retention but also to slow disease progression, treat intercurrent infections, enable timely initiation on ART for those eligible, reduce early mortality after starting ART and thus prolong overall survival.

Our findings should be interpreted in the light of several limitations. First, more than a third of the immunological data were missing. This may have reduced the power of our study to show an effect of CD4 count on pre-ART LTFU. However, clinical indicators have been found to be equally good as markers of immunosuppression and are, in fact, the most commonly adopted method of assessing for HIV disease severity in resource-limited settings. Our data had almost 90% of the clinical data available, none of which suggested an effect on LTFU.

Secondly, although we used an outcome definition that was empirically defined, this definition was only previously studied among patients on ART. Thus, applying the definition on a pre-ART cohort may be deemed restrictive. The narrow time interval used may have resulted in clients being misclassified as LTFU even when they resumed care later, which may have resulted in an overestimation of the LTFU rate. This limitation implies need for a standardized approach to defining LTFU in the pre-ART population based on empiric evidence.

Lastly, censoring clients who were later enrolled in the AHT/FBP programmes may have biased our findings. A comparison of clients that were enrolled in the AHT/FBP programmes to those that were not suggested these groups had similar baseline characteristics for most variables. However, the mean baseline CD4 count of clients enrolled in the AHT/FBP programmes was higher compared to that of those who were not enrolled. This suggests that censored clients were in fact less immunocompromised, which may have potentially resulted in a shift in the results with an effect being observed on LTFU among healthier clients if they were not enrolled in the AHT/FBP programmes.

In conclusion, so far most attention has been given to LTFU among patients on ART. Our study with recent published data suggests that pre-ART LTFU is a widespread problem in Africa. Importantly, risk factors for pre-ART LTFU are different from those in clients on ART. Our findings warrant consideration of an enhanced pre-ART package aimed at improving retention, care, timely initiation of ART and overall survival.

Further studies are needed to assess the burden and risk factors for pre-ART LTFU in different settings. Cost effectiveness, adherence and side effects of interventions targeted at the pre-ART populations should be assessed to justify their roll out.

## References

[b1] Amuron B, Namara G, Birungi J (2009). Mortality and loss-to-follow-up during the pre-treatment period in an antiretroviral therapy programme under normal health service conditions in Uganda. BMC Public Health.

[b2] Bassett IV, Wang B, Chetty S (2009). Loss to care and death before antiretroviral therapy in Durban, South Africa. Journal of Acquired Immune Deficiency Syndromes.

[b3] Bassett IV, Regan S, Chetty S (2010). Who starts antiretroviral therapy in Durban, South Africa?... not everyone who should. AIDS.

[b4] Boulle A, Bock P, Osler M (2008). Antiretroviral therapy and early mortality in South Africa. Bulletin of the World Health Organization.

[b5] Brinkhof MW, Dabis F, Myer L (2008). Early loss of HIV-infected patients on potent antiretroviral therapy programmes in lower-income countries. Bulletin of the World Health Organization.

[b6] Brinkhof MW, Pujades-Rodriguez M, Egger M (2009). Mortality of patients lost to follow-up in antiretroviral treatment programmes in resource-limited settings: systematic review and meta-analysis. PLoS One.

[b7] Chi BH, Cantrell RA, Mwango A (2010). An empirical approach to defining loss to follow-up among patients enrolled in antiretroviral treatment programs. American Journal of Epidemiology.

[b8] Etienne M, Burrows L, Osotimehin B (2010). Situational analysis of varying models of adherence support and loss to follow up rates; findings from 27 treatment facilities in eight resource limited countries. Tropical Medicine and International Health.

[b9] Fenner L, Brinkhof MW, Keiser O (2010). Early mortality and loss to follow-up in HIV-infected children starting antiretroviral therapy in Southern Africa. Journal of Acquired Immune Deficiency Syndromes.

[b10] Fox MP, Rosen S (2010). Patient retention in antiretroviral therapy programs up to three years on treatment in sub-Saharan Africa 2007–2009: systematic review. Tropical Medicine and International Health.

[b11] Grant AD, Charalambous S, Fielding KL (2005). Effect of routine isoniazid preventive therapy on tuberculosis incidence among HIV-infected men in South Africa: a novel randomized incremental recruitment study. JAMA.

[b12] Karcher H, Omondi A, Odera J, Kunz A, Harms G (2007). Risk factors for treatment denial and loss to follow-up in an antiretroviral treatment cohort in Kenya. Tropical Medicine and International Health.

[b13] Krebs DW, Chi BH, Mulenga Y (2008). Community-based follow-up for late patients enrolled in a district-wide programme for antiretroviral therapy in Lusaka, Zambia. AIDS Care.

[b14] Larson BA, Brennan A, Mcnamara L (2010). Early loss to follow up after enrolment in pre-ART care at a large public clinic in Johannesburg, South Africa. Tropical Medicine and International Health.

[b15] Lawn SD, Harries AD, Anglaret X, Myer L, Wood R (2008). Early mortality among adults accessing antiretroviral treatment programmes in sub-Saharan Africa. AIDS.

[b16] Lessells RJ, Mutevedzi PC, Cooke GS, Newell ML (2011). Retention in HIV care for individuals not yet eligible for antiretroviral therapy: rural KwaZulu-Natal, South Africa. Journal of Acquired Immune Deficiency Syndromes.

[b17] Losina E, Bassett IV, Giddy J (2010). The “ART” of linkage: pre-treatment loss to care after HIV diagnosis at two PEPFAR sites in Durban, South Africa. PLoS One.

[b18] Maskew M, Macphail P, Menezes C, Rubel D (2007). Lost to follow up: contributing factors and challenges in South African patients on antiretroviral therapy. South African Medical Journal.

[b19] McGuire M, Munyenyembe T, Szumilin E (2010). Vital status of pre-ART and ART patients defaulting from care in rural Malawi. Tropical Medicine and International Health.

[b20] Merten S, Kenter E, Mckenzie O, Musheke M, Ntalasha H, Martin-Hilber A (2010). Patient-reported barriers and drivers of adherence to antiretrovirals in sub-Saharan Africa: a meta-ethnography. Tropical Medicine and International Health.

[b21] Ochieng-Ooko V, Ochieng D, Sidle JE (2010). Influence of gender on loss to follow-up in a large HIV treatment programme in western Kenya. Bulletin of the World Health Organization.

[b22] Rosen S, Fox MP, Gill CJ (2007). Patient retention in antiretroviral therapy programs in sub-Saharan Africa: a systematic review. PLoS Med.

[b23] Russell EC, Charalambous S, Pemba L, Churchyard GJ, Grant AD, Fielding K (2010). Low haemoglobin predicts early mortality among adults starting antiretroviral therapy in an HIV care programme in South Africa: a cohort study. BMC Public Health.

[b24] Tassie JM, Baijal P, Vitoria MA, Alisalad A, Crowley SP, Souteyrand Y (2010). Trends in retention on antiretroviral therapy in national programs in low-income and middle-income Countries. Journal of Acquired Immune Deficiency Syndromes.

[b25] Toure S, Kouadio B, Seyler C (2008). Rapid scaling-up of antiretroviral therapy in 10,000 adults in Cote d’Ivoire: 2-year outcomes and determinants. AIDS.

[b26] UNAIDS (2010). UNAIDS Outlook Report.

[b27] Walson JL, Otieno PA, Mbuchi M (2008). Albendazole treatment of HIV-1 and helminth co-infection: a randomized, double-blind, placebo-controlled trial. AIDS.

[b28] WHO (2006). Antiretroviral Therapy for HIV Infection in Adults and Adolescents: Recommendations for a Public Health Approach.

[b29] Yiannoutsos CT, An MW, Frangakis CE (2008). Sampling-based approaches to improve estimation of mortality among patient dropouts: experience from a large PEPFAR-funded program in Western Kenya. PLoS One.

[b30] Yu JK, Chen SC, Wang KY (2007). True outcomes for patients on antiretroviral therapy who are “lost to follow-up” in Malawi. Bulletin of the World Health Organization.

